# Transcranial magnetic stimulation for tinnitus: using the Tinnitus Functional Index to predict benefit in a randomized controlled trial

**DOI:** 10.1186/s13063-017-1807-9

**Published:** 2017-02-09

**Authors:** Sarah M. Theodoroff, Susan E. Griest, Robert L. Folmer

**Affiliations:** 1 VA RR&D, National Center for Rehabilitative Auditory Research, VA Portland Health Care System, 3710 SW US Veterans Hospital Road (NCRAR – P5), Portland, OR 97239 USA; 2 Department of Otolaryngology, Head-Neck-Surgery, Oregon Health and Science University, 3181 SW Sam Jackson Park Road, Portland, OR 97239 USA

**Keywords:** Tinnitus, Transcranial magnetic stimulation, Questionnaire

## Abstract

**Background:**

Identifying characteristics associated with transcranial magnetic stimulation (TMS) benefit would offer insight as to why some individuals experience tinnitus relief following TMS treatment, whereas others do not. The purpose of this study was to use the Tinnitus Functional Index (TFI) and its subscales to identify specific factors associated with TMS treatment responsiveness.

**Methods:**

Individuals with bothersome tinnitus underwent 2000 pulses of 1-Hz TMS for 10 consecutive business days. The primary outcome measure was the TFI which yields a total score and eight individual subscale scores. Analyses were performed on baseline data from the active arm (*n* = 35) of a prospective, double-blind, randomized placebo-controlled clinical trial of TMS for tinnitus.

**Results:**

Baseline total TFI score and three of the eight TFI subscales were useful in differentiating between responders and nonresponders to TMS intervention for tinnitus. These findings are not definitive, but suggest potential factors that contribute to perceived benefit following TMS.

**Conclusions:**

Overall, the main factor associated with TMS benefit was a higher tinnitus severity score for responders at baseline. The TFI subscales helped to clarify the factors that contributed to a higher severity score at baseline. Large-scale prospective research using systematic approaches is needed to identify and describe additional factors associated with tinnitus benefit following TMS.

**Trial registration:**

ClinicalTrials.gov, ID: NCT01104207. Registered on 13 April 2010.

## Background

Measures of tinnitus distress or severity are often used to evaluate to what degree patients benefit from an intervention. Unfortunately, the majority of instruments designed to measure tinnitus severity were not developed to assess treatment outcomes [1]. Meikle et al. [2] discuss the distinction between outcome measures designed for screening purposes versus measures designed to evaluate treatment responsiveness; that is, to detect improvement over time. It is essential that the outcome measures accurately assess the tinnitus severity (validity) and do so with minimum error (reliability). Ideally, clinicians use an evidence-based approach in making a decision regarding the best method to assess tinnitus therapy. Following the model of evidence-based medicine allows clinicians to integrate their clinical expertise with evidence from systematic research to guide their decision-making on how best to evaluate and treat patients. However, evidence is lacking regarding the best way to assess and treat tinnitus. Therefore, at the current time, clinicians must rely mainly on their clinical experiences and judgment regarding the best course of action to take when evaluating and treating tinnitus patients.

Tinnitus therapy offers hope for patients by acknowledging that their problem is not only “in their head” and it is possible that something can be done to evaluate and treat their condition. For many patients, any claim of an effective tinnitus intervention, regardless of the evidence, is worth pursuing. Unfortunately, there are many experimental “treatments” promoted as effective but without supporting evidence [3]. Well-designed clinical trials demonstrating treatment efficacy need to be conducted in order for these claims to be substantiated. This issue raises the question of how best to determine treatment effectiveness.

The Tinnitus Functional Index (TFI) is a relatively new questionnaire, developed by Meikle et al. [4], with the concept of responsiveness central to its design. The TFI is a 25-item questionnaire that has good content validity in terms of treatment responsiveness as well as improved sensitivity for assessing tinnitus severity. The TFI has a 0-to-10 response format for each question, allowing for finer scaling of tinnitus severity (total possible TFI score ranges from 0 to 100). Another advantage of the TFI is its ability to evaluate a broad array of tinnitus-related problems by having a total of eight subscales: Intrusive, Sense of Control, Cognitive, Sleep, Auditory, Relaxation, Quality of Life, and Emotional.

The TFI has been validated psychometrically, showing high internal consistency (Cronbach’s *α* = 0.98) and high test-retest reliability (*r* = 0.91). Internal consistency and test-retest reliability were also high for each of the eight subscales with values ranging from *α* = 0.87–0.97 and *r* = 0.71–0.92, respectively [4]. For additional information on the design and suggested use of the TFI questionnaire, see Meikle et al. [4].

The aim of this substudy is to use the TFI to identify patient factors associated with responsiveness to transcranial magnetic stimulation (TMS) treatment for tinnitus. Mennemeier et al. [5] state the importance of investigating factors associated with TMS treatment benefit to advance the development of TMS as a treatment for tinnitus. TMS has the potential to become an effective clinical intervention for tinnitus sufferers. Identifying factors associated with TMS responsiveness would allow the treatment to be targeted to individuals most likely to experience benefit. The purpose of this substudy is to use the TFI and its subscales to identify factors across multiple domains of tinnitus severity that are associated with patients’ responsiveness to TMS treatment for tinnitus.

## Methods

Data reported and analyzed in this substudy are from a prospective, double-blind, randomized placebo-controlled clinical trial of repetitive TMS for tinnitus treatment [6].

### Clinical trial methods

In the clinical trial, 70 subjects were randomly assigned to receive either active or placebo TMS daily for 10 consecutive business days. Whether subjects received the TMS procedure on the left or right side of the head was also randomized. During each session of TMS, 2000 pulses of TMS were administered at a rate of 1 Hz. Assessments were performed at baseline, immediately following the last (10th) TMS session, and 1, 2, 4, 13, and 26 weeks thereafter. The primary outcome measure was the TFI.

### Responder versus nonresponder

To determine whether a clinically significant change occurred, TFI scores were examined immediately following the 10th TMS session (i.e., post TMS) and compared to baseline scores. An improvement of >7 TFI points was used to classify a “responder” [6]. Using this criterion, 18 out of 35 subjects in the active TMS group were classified as responders and 9 out of 35 subjects in the placebo TMS group were classified as responders.

The placebo TMS coil used in the clinical trial produced sounds and sensations similar to the active TMS coil. To better mimic the sounds and scalp sensations perceived by subjects in the active TMS group, the placebo coil in the clinical trial was set to a relatively high intensity, on average 60.8%. This stimulation intensity makes it possible that our placebo coil was not completely “inert.” The placebo coil contains a metal plate that supposedly interferes with the usual generation and transmission of the magnetic field to the subjects’ brain. Additional information about the placebo group and other findings from the randomized clinical trial are reported by Folmer et al. [6]. For this substudy, results were calculated from baseline data comparing responders to nonresponders.

### Methods: substudy

Subjects in the current substudy included 28 men and 7 women ranging in age from 32 to 73 years (mean = 58.5; *SD* = 9.3). Only subjects randomized to the active arm of the clinical trial were included (*n* = 35). To be consistent with the clinical trial, the TFI was the primary outcome measure used in this substudy. The criterion established in the clinical trial for responders and nonresponders to the TMS intervention was used in the current substudy. The mean age of responders (*n* = 18) was 56.7 (*SD* = 10.9 years) and 60.5 (*SD* = 7.0 years) for nonresponders (*n* = 17). No statistically significant differences were found in age between responders and nonresponders.

### Hearing ability

All degrees of sensorineural hearing loss were included in the current substudy. Individuals with significant hearing loss who are bothered by their tinnitus are an important subpopulation because often they do not benefit from acoustic therapy to manage their tinnitus and are, therefore, in need of an intervention (e.g., TMS) that is not dependent on hearing ability.

### Tinnitus characteristics

All subjects had constant, nonpulsatile tinnitus, of at least 1 year in duration, which was self-rated at a loudness of 6 or greater on a 0-to-10 visual numeric scale. Laterality of tinnitus perception was not correlated with TMS outcome (i.e., responder versus nonresponder) nor with the TMS coil placement (i.e., left side versus right side). Of the 18 subjects who responded to TMS, perceived tinnitus location was reported as: in both ears (*n* = 13), in the head (*n* = 2), dominant in the right ear (*n* = 3), and dominant in the left ear (*n* = 0); of the 17 subjects who were classified as nonresponders, perceived tinnitus location was reported as: in both ears (*n* = 12), in the head (*n* = 2), dominant in the right ear (*n* = 1), and dominant in the left ear (*n* = 2).

### Data analysis

The data analyzed in this substudy were collected as part of the parent clinical trial; therefore, no additional power analysis was performed [6]. Comparisons were made between responders and nonresponders using independent *t* tests. TFI total score and all eight subscale scores were examined. To minimize the risk of multiple *t* tests (nine in total) resulting in observing at least one significant result due to chance, we applied the Bonferroni correction and adjusted the significance level to account for this concern (*p* ≤ 0.01). Statistical analyses were conducted using the Statistical Package for Social Sciences Version 22.

## Results

**Fig. 1:**
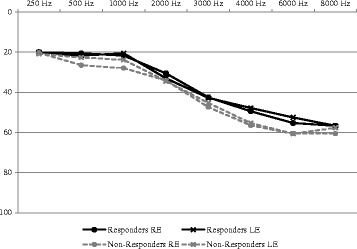
Mean audiograms for responders and nonresponders. Legend: *Solid black lines with circles* represent responders’ right ear mean audiometric thresholds; *solid black lines with “x”* represent responders’ left ear mean audiometric thresholds; *dotted gray lines with circles* represent nonresponders’ right ear mean audiometric thresholds; *dotted gray lines with “x”* represent nonresponders’ left ear mean audiometric thresholds

Table 1 displays the mean hearing thresholds for responders compared to nonresponders and the mean audiograms are displayed in Fig. 1. There were no statistically significant differences in pure tone thresholds between these groups for any test frequencies.

**Table 1:** Mean hearing thresholds (dB Hearing Level (dB HL)) for responders and nonresponders

Right ear (Hz)	250	500	1000	2000	3000	4000	6000	8000
Responders	20.00 (9.07)	20.56 (11.10)	21.67 (13.93)	30.56 (19.77)	42.50 (22.64)	49.44 (22.02)	55.28 (21.59)	56.67 (22.62)
Nonresponders	20.29 (9.10)	26.47 (16.28)	27.94 (18.96)	34.12 (21.30)	47.35 (22.78)	56.47 (21.63)	60.59 (21.71)	60.59 (20.61)
Left ear (Hz)	250	500	1000	2000	3000	4000	6000	8000
Responders	20.00 (9.39)	21.67 (11.50)	20.56 (13.27)	33.06 (20.23)	42.78 (22.24)	47.78 (20.16)	52.50 (18.65)	56.67 (22.94)
Nonresponders	20.88 (9.72)	22.65 (10.91)	23.82 (12.81)	34.41 (16.00)	45.29 (19.88)	55.29 (21.83)	60.59 (19.03)	57.65 (16.97)

Table 2 displays results and statistical analyses for responders compared to nonresponders at baseline on the total TFI score and all TFI subscale scores. Of the eight TFI subscales, the Auditory TFI subscale revealed a statistically significant difference for responders compared to nonresponders, with responders showing higher scores at baseline (*p* = 0.007); two other TFI subscales approached statistical significance: Cognitive (*p* = 0.022) and Emotional (*p* = 0.023) again showing higher severity scores at baseline. TFI total score also approached significance with responders having higher TFI total scores at baseline (*p* = 0.017).

**Table 2:** TFI mean scores and independent *t t*est results

TFI at baseline		Mean (*SD*)	Significance (2-tailed)
Total score	Responder	51.38 (18.69)	0.017
	Nonresponder	36.73 (15.66)	
Intrusive subscale	Responder	68.15 (17.65)	0.193
	Nonresponder	60.20 (17.74)	
Sense of Control subscale	Responder	62.59 (22.04)	0.223
	Nonresponder	52.94 (23.98)	
Cognitive subscale	Responder	46.67 (25.08)	0.022
	Nonresponder	26.86 (23.83)	
Sleep subscale	Responder	47.04 (28.21)	0.049
	Nonresponder	27.65 (28.01)	
Auditory subscale	Responder	60.00 (15.88)	0.007^a^
	Nonresponder	37.65 (27.02)	
Relaxation subscale	Responder	55.00 (28.43)	0.331
	Nonresponder	46.27 (23.54)	
Quality of Life subscale	Responder	38.19 (24.14)	0.119
	Nonresponder	25.44 (22.87)	
Emotional subscale	Responder	37.78 (25.13)	0.023
	Nonresponder	20.59 (16.72)	

^a^ *p* ≤ 0.01; *TFI* Tinnitus Functional Index, *SD* Standard deviation

## Discussion

The primary aim of this substudy was to use the TFI and its subscales to identify specific factors associated with TMS treatment-responsiveness for tinnitus. In general, responders had higher overall tinnitus severity scores at baseline compared to nonresponders. A higher baseline severity score among treatment responders is consistent with the findings of Lehner et al. [7]. Using a different outcome measure of tinnitus severity, the Tinnitus Questionnaire (TQ), Lehner et al. [7] reported that subjects with higher TQ baseline scores showed greater improvement following TMS treatment for tinnitus compared to subjects with lower TQ baseline scores prior to treatment. Both the current substudy and Lehner et al. [7] showed baseline levels of tinnitus severity to be associated with TMS treatment responsiveness.

One advantage of using the TFI as the primary outcome measure is that, of all the available tinnitus questionnaires, the TFI was specifically designed to measure treatment responsiveness [4, 8]. The TFI is highly correlated with the Tinnitus Handicap Inventory, but for the purposes of identifying factors associated with treatment *responsiveness*, it is imperative to use an instrument developed and designed to specifically address this psychometric property [1, 4].

Examining the TFI subscales allows for a greater understanding of the factors associated with the effectiveness of specific tinnitus interventions [4]. The Auditory TFI subscale showed significant differences between responders and nonresponders at baseline and assesses perceptual difficulties individuals attribute to be related to tinnitus. Specifically, questions in this subscale assess the degree to which individuals believe that their tinnitus interferes with their ability to hear, to understanding what people are saying, and to follow the thread of a conversation. By assessing a tinnitus patient’s ability to follow conversations, the Auditory TFI subscale could be indirectly sensitive to attentional problems. The Cognitive TFI subscale is directly sensitive to attentional-related problems associated with tinnitus such as the ability to concentrate, to think clearly, and to focus attention away from tinnitus. Both the Auditory and Cognitive TFI subscales showed that responders to TMS had higher severity scores on these subscales at baseline.

It is not uncommon for people with tinnitus and hearing loss to attribute communication problems solely to their tinnitus. In the current substudy, all subjects had hearing loss (see Fig. 1). It is sometimes difficult for tinnitus patients to distinguish tinnitus-related problems from hearing-related problems. When these conditions co-occur, tinnitus is frequently “blamed” for the communication problems when, more than likely, the underlying hearing loss is more of a contributing factor [9–11]. Tinnitus-related problems are often intertwined with hearing-related problems, making it difficult to isolate one issue from the other. Henry et al. [12] addressed this dilemma by developing the Tinnitus and Hearing Survey, a brief questionnaire that is statistically validated for differentiating tinnitus-related problems from hearing-related problems. The Tinnitus and Hearing Survey can be used as a counseling tool and can assist the clinician in discussing with patients what appears to be impacting daily functioning (e.g., tinnitus- versus hearing-related problems).

The TFI Emotional subscale also exhibited meaningful differences between responders and nonresponders at baseline. This subscale evaluates the degree to which individuals feel anxious, bothered, and depressed because of their tinnitus. People who have bothersome tinnitus sometimes exhibit anxiety symptoms that may or may not be clinically significant to the degree of qualifying as an anxiety disorder. Severe tinnitus is often associated with emotional distress, showing high correlations with anxiety and depression [13].

Because TMS stimulates cortical structures, using TMS to treat tinnitus might also result in other clinical benefits. TMS produces a magnetic field which induces an electric current in underlying neural tissue and ultimately affects neuronal activity of structures in targeted areas [14]. It is possible that, in addition to targeted cortical structures, other neuronal networks, such as those involved with attention, cognition, or auditory processing, might also be affected during this process.

## Conclusions

Differential effects of TMS for treatment of tinnitus have been reported in multiple studies [15–17]. Findings from the current substudy, along with results from Lehner et al. [7], suggest that the degree of tinnitus severity at baseline is associated with TMS treatment responsiveness. The TFI subscales offer valuable information and point to specific factors that contribute to responding to an intervention.

It is important to remember that this substudy recruited individuals who had bothersome tinnitus. Examining overall TFI score at baseline is a good starting point, but there is not an established “cutoff” score that separates individuals in need of help from those who do not. In general, a more severe baseline score means an individual has more room to improve following any form of intervention or counseling. How we determine how much improvement following an intervention results in a clinically significant change is a different matter.

Defining what “clinically significant change” is in terms of treatment effects is not straightforward. Multiple methods have been proposed addressing the concept of clinical significance. Jacobson et al. [18] discuss various methods for defining clinical significance and state, “Clinical significance is routinely defined as returning to normal functioning. Although for some disorders this may be too stringent a criterion, it is based on the assumption that consumers enter therapy expecting that their presenting problems will be solved.” (p. 300).

A caveat when applying this to tinnitus interventions is that widespread acceptance regarding what degree or type of change is consistent with clinically significant improvement does not exist. This quandary is not isolated to tinnitus or the field of audiology. In the field of psychology, Kazdin [19] puts forth the idea that clinical significance can be interpreted in many different ways. Kazdin makes an important point that the problem and goals of treatment are influencing factors. Regarding tinnitus interventions, some goals focus on changing characteristics of the tinnitus perception (e.g., pitch, loudness) and other goals are directed at reducing tinnitus-related distress, without any change in the tinnitus perception itself. Therefore, there are a multitude of possible changes following tinnitus interventions that can occur, all of which can contribute to whether or not clinically significant change has occurred.

Tinnitus is complex and cannot be directly measured. Therefore, it is challenging to define benefit in terms of “solving the problem” because for some individuals the expectation is eliminating the tinnitus (i.e., finding a cure) and for others it is obtaining some form of symptomatic relief, but not necessarily quieting the tinnitus. An advantage of using the TFI is the ability of its subscales to explain what “relief” looks like from the individuals’ point of view in terms of improvement post intervention.

This substudy used the TFI to identify patient factors associated with responsiveness to TMS for tinnitus. Examining the differences between responders and nonresponders at baseline provides insight into possible factors associated with benefit using TMS for tinnitus. Specifically, for this sample population, it suggests that factors other than the tinnitus characteristics (e.g., loudness) as captured by the elements on the Auditory, Cognitive, and Emotional TFI subscales had an important influence on TMS outcome.

### Limitations of current study

Due to the small sample size, the current study is limited in terms of conducting complex data analyses. Preliminary results are compelling and, therefore, prospective research using systematic approaches and larger numbers of subjects is needed to identify predictive as well as other factors that contribute to differentiating patient responsiveness to TMS as an intervention for tinnitus. Conducting these future studies is essential to maximize the efficacy of TMS for tinnitus and to determine if certain characteristics or factors would identify certain individuals as ideal candidates for responding to TMS for tinnitus.

## Abbreviations

TFI: Tinnitus Functional Index
TMS: Transcranial magnetic stimulation
TQ: Tinnitus Questionnaire
